# Comparison of Gene-expression Profiles between Normal and Periodontitis-affected Tissues

**Published:** 2017-05

**Authors:** So-Youn AN, Ki Seok HONG, Jong-Tae PARK

**Affiliations:** 1. Dept. of Pediatric Dentistry, College of Dentistry, Wonkwang University, Daejeon, South Republic of Korea; 2. Perio-Implant Research Center, Dankook University College of Dentistry, Cheonan, South Republic of Korea; 3. Dept. of Oral Anatomy, Dankook University College of Dentistry, Cheonan, South Republic of Korea

## Dear Editor-in-Chief

Periodontitis is an inflammatory disease that affects the periodontium caused by multiple factors such as bacterial biofilms, smoking, and diabetes (1). The differential expression of several genes was associated with periodontitis, such as those encoding interleukin (IL; *IL*), vascular endothelial growth factor (*VEGF*), and intercellular adhesion molecule (*ICAM*), using previously published microarray results (2). Long noncoding RNAs could be influenced and play an important role in the pathogenesis of periodontitis, and potential targets for the future treatment of periodontitis and as novel diagnostic biomarkers for periodontitis have been suggested (3). However, most of the relevant research has thus far focused on the immune response and differences in gene-expression patterns between normal and periodontitis-affected tissues that are difficult to detect. Therefore, the purpose of this study was to compare gene-expression profiles between healthy and CP gingival tissues.

This study was performed at the Department of Periodontology, School of Dental Medicine, Dankook University, Korea, and was approved by the Institutional Review Board and the Ethic Committee of Dankook University Dental Hospital (IRB No. H1406/008/002). All of the involved patients provided informed consent for their tissues to be used in this study.

The age of the patients was 40–60 yr. Tissues of patients who had probing depth over 6 mm were used in the study.

**Fig. 1:**
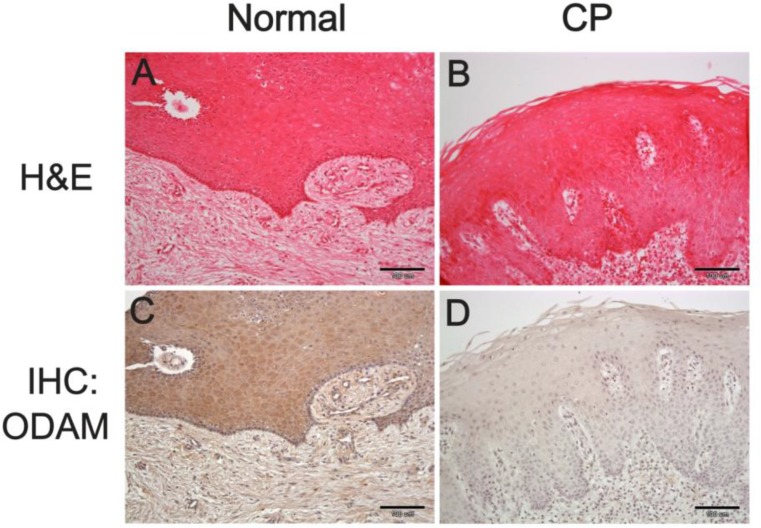
Immunohistochemistry (IHC) staining of ODAM protein from normal and CP tissue. (A) H&E staining of normal tissue. (B) H&E staining of CP tissue. (C) ODAM protein expression in normal tissue. (D) ODAM protein expression in CP tissue. Scale bar : 100 μm

The histological characteristics of CP tissues were determined by performing H&E and IHC staining. Isolated gingiva samples were fixed overnight using 4% paraformaldehyde at 4 °C, embedded in paraffin, sectioned, and stained with hematoxylin and eosin (H&E) or subjected to immunohistochemistry (IHC) staining. The mouse control sections and antirabbit ODAM antibody were provided. Briefly, the sections were incubated overnight at 4 °C with antirabbit ODAM antibody, and with secondary anti-rabbit IgG antibody at room temperature for 30 min. They were reacted with avidin-biotin-peroxidase complex (Vector Laboratory, Burlingame, CA, USA). Antibody signals were converted using a diaminobenzidine kit, and cell nuclei were stained with hematoxylin. The H&E staining revealed that inflammatory cell penetration was more severe in CP tissue than in its healthy (normal, Fig. 1A) counterpart (Fig. 1B). In IHC, ODAM protein expression was detected in normal JE (Fig. 1C), but ODAM protein was not detected in CP (Fig. 1D). FDCSP and ODAM are known to be junctional epithelium (JE) markers (4). The expressions of *FDCSP* and *ODAM* in normal and CP samples were analyzed using reverse-transcription PCR and real-time PCR.

**Fig. 2:**
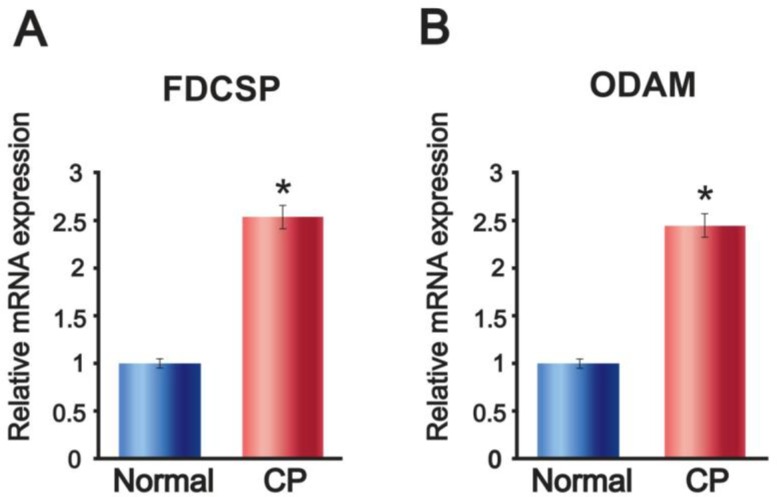
Polymerase-chain-reaction amplification of the genes encoding follicular dendritic-cell-secreted protein (*FDCSP*) and odontogenic ameloblast-associated protein (*ODAM*). mRNA was isolated from normal and CP tissues. The expressions of *FDCSP* and *ODAM* were increased in chronic periodontitis (CP) compared to normal tissue. (A) *FDCSP* mRNA expression. (B) *ODAM* mRNA expression. Data are the mean ± SD of triplicate experiments. * mean data is significantly different from control (*P*<0.01)

As expected, the mRNA expressions of *FDCSP* and *ODAM* were increased in CP compared to normal tissues (Fig. 2).

All quantitative data are presented as the mean±SD. Statistical differences were analyzed using Student’s *t*-tests (*P*<.01). Therefore, progression of periodontitis induces the expression of FDCSP and ODAM.

Periodontitis is an inflammatory disease with a variety of etiologies, including bacterial lipopolysaccharide (LPS). During the progression of periodontitis, gene-expression patterns change rapidly and biological pathways are immediately activated in attempts to prevent infection and restore the damaged tissues (5). In this study, *ODAM* mRNA expression was increased, while IHC analysis revealed no ODAM protein in CP tissue. Recently, destruction of the PDL integrity elicits the expression of *ODAM* in the ERM (6). ODAM participates in maintaining the integrity and homeostasis of the JE and PDL during the early stages of periodontitis. However, ODAM protein was not detected in migrating epithelial cells because of released to GCF pocket (7).

The levels of *FDCSP* and *ODAM* mRNA appear to be increased in periodontitis. FDCSP and ODAM play important roles in the progression of this condition. Further investigation is required to identify the relationship between FDCSP and periodontitis.
